# Aerobic capacity, strength, and cognitive performance of e-sports athletes and their determinants

**DOI:** 10.3389/fspor.2026.1940173

**Published:** 2026-09-18

**Authors:** Fernanda Patti Nakamoto, Alyne Korukian Freiberg, Marcus V. L. dos Santos Quaresma, Hillary Chrysti de Almeida, Rachel Vaini Barczyszyn, Matheus Pio dos Santos, Eduardo Saucedo Lage, Raphael Einsfeld Simões Ferreira, Luiza Helena Degani-Costa

**Affiliations:** 1 Exercise and Quality of Life Research Laboratory, Centro Universitário São Camilo, São Paulo, Brazil; 2 Department of Pediatric Pulmonology, University of São Paulo, São Paulo, Brazil; 3 Faculdade Israelita de Ciências da Saúde Albert Einstein—Hospital Israelita Albert Einstein, São Paulo, Brazil

**Keywords:** cognition, e-sports, performance, physical conditioning, training

## Abstract

**Aim:**

To characterize the physical and cognitive performance of professional e-sports athletes (E-SG) and compare them with healthy university students (CG).

**Methods:**

Physical performance was assessed cross-sectionally by handgrip strength (HGS) and % predicted V̇O_2_ at peak and anaerobic threshold (V̇O_2_peak% and V̇O_2_AT%) of an incremental cardiopulmonary exercise test. Computer-based tests were used to evaluate memory and reaction time. Using a comprehensive health assessment, we investigated the determinants of physical and cognitive performance and how they related to ranking at the most recent championship.

**Results:**

Sixty-four male participants (34 E-SG; 30 CG) were included. Age, body composition, and blood test results did not differ between the groups. E-SG demonstrated higher sedentary time (*p* < 0.001), reduced HGS (47.23 ± 8.69 kgf vs. 42.8 ± 8.88 kgf; *p* = 0.027), and worse V̇O_2_peak% compared to CG (106.7 ± 13.49 vs. 86.4 ± 12.05; *p* < 0.001) and V̇O_2_AT% (86.00 ± 13.35 vs. 68.5 ± 13.56; *p* < 0.001). Being an E-SG and having lower fat-free mass were independent predictors of lower V̇O_2_peak%, while sedentary time was inversely associated with V̇O_2_AT%. E-SG displayed faster working memory (736 ms vs. 893 ms; *p* = 0.016), which showed a small but positive association with championship rank (*β* = 0.076; *p* = 0.011). Training time was a borderline predictor of ranking.

**Conclusions:**

Professional e-sports athletes presented reduced aerobic fitness, high sedentary behavior, and faster working memory compared to healthy university students, highlighting the complex interplay between professional needs and their potential health impacts.

## 1 Introduction

In recent years, electronic sports (e-sports) have emerged as a field in which digital gaming culture intersects with traditional sports. Advances in technology and digital platforms have facilitated the global expansion of e-sports, which has become a multibillion-dollar industry.

As with traditional competitive sports players, a predefined set of skills for e-sports players should be sought through extensive training. Each type of game has different demands on players' skills, but most require precision and reaction speed, with milliseconds often determining the outcome (1). Indeed, e-sports athletes simultaneously engage cognitive and motor skills to optimize decision-making and reaction times during gameplay (2). Studies indicate that gamers generally outperform non-gamers in visual reaction time and excel in visual memory tasks, including sequence and immediate memory tasks.

Competitive e-sports players typically engage in training for 5.5 to 10 h each day, often remaining seated continuously for up to 3 h or more. Elite competitors may practice for over 10 h daily, performing repetitive and precise movements with a mouse and keyboard, sometimes executing as many as 400 fine motions per minute (1). Nevertheless, prolonged sedentary behavior, low physical activity, lengthy screen time, repetitive movements, and perhaps concurrent unhealthy habits/lifestyle among e-sports players can have significant effects on both their physical and mental health.

It has been shown that e-sports athletes are less physically fit and often develop eye strain, repetitive strain injuries (e.g., tendinitis, carpal tunnel syndrome), sleep disorders, and mood disturbances (3), which might be directly related to the lifestyle and daily routine they adopt. In addition to potential long-term adverse health consequences, these unwanted conditions may also undermine their training and negatively affect their performance in e-sports.

Therefore, the primary aim of this study was to characterize physical performance (strength and cardiopulmonary fitness) and cognitive performance (memory and reaction times) of competitive e-sports athletes, comparing them to healthy university students who did not compete in e-sports. Secondarily, we sought to investigate the determinants of physical and cognitive performance and explore how they related to the professional achievements of the e-sports athletes in their most recent championship.

## 2 Methods

### 2.1 Study type, ethical approval, and informed consent

In this quantitative cross-sectional study, Brazilian professional e-sports athletes and matched university students were invited to undergo a comprehensive assessment at the Exercise and Quality of Life Research Laboratory of São Camilo University Center (São Paulo, Brazil). Following approval by the local ethics committee (n◦ 6.658.033), data were collected between February 2024 and June 2025. All volunteers signed the informed consent form and the study was conducted in accordance with the precepts of the Declaration of Helsinki (4).

### 2.2 Participants and recruitment strategies

We included international-level professional e-sports athletes who competed in three categories: Multiplayer Online Battle Arenas (MOBA), First-Person Shooters (FPS), and Squad Battle Royale. Athletes were recruited by directly contacting team managers. Age-, sex-, and body mass index (BMI)-matched university students were invited to participate in the control group and were recruited via social media and advertisements on university campuses.

The study was originally designed to include both male and female participants, but the professional e-sports teams that agreed to collaborate with our study were exclusively composed of male players; therefore, only male controls were recruited.

Volunteers from both groups were systematically screened for contraindications to performing maximal exercise testing. Individuals with ongoing orthopedic or muscle injuries, uncontrolled hypertension or arrhythmias, as well as those with significant or uncontrolled cardiopulmonary or neuromuscular diseases that could potentially impair aerobic capacity or place the subject under increased risk were excluded. Obesity alone was not considered an exclusion criterion.

### 2.3 Instruments and procedures

During a single study visit, participants systematically underwent five different procedures: i) completion of a comprehensive health questionnaire; ii) anthropometric measurement and body composition assessment; iii) computer-based memory and reaction time tests; iv) cardiopulmonary exercise testing (CPET) and handgrip strength test; and v) biochemical profiling.

All scheduled assessments should occur during a championship season, with a small window of tolerance of up to two weeks before or after the championship being accepted. Professional performance (vi) on that given championship was later assessed.

#### 2.3.1 Questionnaire

An electronic questionnaire comprising 66 multiple-choice questions divided into three sections was completed using REDCap (Supplementary Material). Section A (Demographics) investigated the sociodemographic profile, including age, sex, gender, family income, comorbidities, and modality of the e-sports played (when applicable). Section B (Mental Health) gathered information about the use of legal (tobacco, alcohol) and illegal substances (electronic cigarettes, cannabis, cocaine, MDMA, and stimulants), quantified weekly caffeine intake, and included anxiety and depression screening tools (Hospital Anxiety and Depression Scale [HADS] (5) and Patient Health Questionnaire-9 [PHQ-9] (6), respectively). Finally, Section C (Physical Activity, Sleep, and Quality of Life) assessed the level of physical activity and overall sedentary time during a typical week (7, 8), sleep quality and efficiency using the Pittsburgh Sleep Quality Index (PSQI) (9, 10), and perceived quality of life using the European Quality of Life 5 Dimensions 3 Level Version (EQ-5D-3L) questionnaire (11). All standardized screening tools included in the study had been translated, culturally adapted, and validated for the Brazilian population.

#### 2.3.2 Anthropometric measurements and body composition

Weight and height were measured (12), followed by bioelectrical impedance analysis of body composition (BIA; Biodynamics® model 450) (13). All assessments were conducted after at least four hours of fasting.

#### 2.3.3 Neurocognitive tests

Following a light snack given immediately after BIA, the volunteers underwent five computer-based neurocognitive tests using the Ubrain® online platform, always in the following order: i. Episodic Memory Test; ii. Simple Reaction Test; iii. Choice Reaction Test; iv. Verbal Memory Test; v. Working Memory Test. Participants received written and verbal instructions on what each test consisted of and were granted a 30 s training session before each test to familiarize themselves with the type of assessment beforehand. Tests generally involved showing an image on a computer screen (playing cards, words, or a target, depending on the test) and asking participants to take some specific action using the keyboard or computer mouse, assessing how fast and how accurately the participants responded (Supplementary material).

#### 2.3.4 Physical fitness and strength assessment

Physical fitness was assessed using an incremental cardiopulmonary exercise test (CPET) performed on a treadmill (Super ATL®, Inbrasport, Porto Alegre, RS, Brazil) until the limit of tolerance. The incremental phase consisted of progressive increases in speed and slope which were tailored to resemble a ramp increment and allow exercise to last 8 to 12 min (14). Breath-by-breath gas exchange was measured using a metabolic system (K5, COSMED, Rome, Italy), along with beat-by-beat heart rate monitoring (GARMIN® chest strap). Peripheral oxygen saturation was assessed using a pulse oximeter (NONIN Onyx Vantage®).

Handgrip strength (HGS) was assessed using a hydraulic hand dynamometer (JAMAR®). The participants performed HGS tests with both hands three times each, with a 30 s rest period between contractions. For data analysis, the highest value obtained was recorded. Both HGS and V̇O_2_peak were compared with age- and sex-predicted values (15, 16).

#### 2.3.5 Biochemical analysis

Blood samples were sent for complete blood cell count, glycated hemoglobin, iron, ferritin, thyroid-stimulating hormone (TSH), thyroxine (T4), vitamin B12, and vitamin D. All analyses were performed at São Camilo Hospital, following their standard procedures.

#### 2.3.6 Professional performance

We identified the championship that was closest to the subjects' study visit and analyzed different scores depending on the e-sports category and game. For Multiplayer Online Battle Arena (MOBA)–League of Legends (MOBA-LOL), we analyzed *Kill Death Assists* (*KDA*); for First Person Shooter—Valorant (FPS—Valorant), we analyzed *Kills, Assists, Survived, and Traded* (*KAST*); for First Person Shooter—Rainbow Six (FPS—R6), we analyzed *Rating*; and for Squad Battle Royale—Free Fire (Battle Royale—FF), we analyzed *Kills* to measure success. Publicly available championship results were used to compare our subjects' scores to those of other athletes in the same championship, determining the subjects' ranking in percentile terms. For MOBA—LOL, comparison also considered the position the athletes held in the game.

### 2.4 Statistical analysis

All statistical analyses were performed using Jamovi software (version 2.6, The Jamovi Project, Sydney, Australia). Statistical significance was set at *p* ≤ 0.05 for two-sided tests. Continuous variables were assessed for normality using the Shapiro–Wilk test and were expressed as mean and standard deviation or median and interquartile range, as appropriate. Between-group comparisons were performed using independent-samples *t*-tests or Welch's *t*-tests when homogeneity of variances was violated, and Mann–Whitney *U*-tests for non-normally distributed variables. Categorical variables were expressed as absolute and relative frequencies and compared using chi-square or Fisher's exact tests, as appropriate.

No formal *a priori* sample size or power calculation was performed. This study was designed as an exploratory cross-sectional investigation of professional e-sports athletes, and recruitment was primarily determined by the availability of eligible professional players and the feasibility of completing the extensive physiological, neurocognitive, and questionnaire-based assessment protocol. Accordingly, no formal adjustment for multiple comparisons was applied, and the regression analyses should be considered exploratory, intended to identify potential associations rather than to provide confirmatory estimates of independent effects.

Multivariable linear regression models were fitted for handgrip strength, V̇O_2_peak (mL·kg^−1^·min^−1^), percentage of predicted V̇O_2_peak, percentage of predicted V̇O_2_peak at the anaerobic threshold and working memory performance. Candidate predictors were defined *a priori* based on biological plausibility, empirical evidence, and their potential role as confounding factors. Model fit was evaluated using *R*^2^, adjusted *R*², and the Akaike Information Criterion (AIC), along with the incremental variance explained by additional covariates. Further details of the statistical analysis are provided in the Supplementary Material.

## 3 Results

A total of 92 participants were assessed for eligibility. Of these, five were excluded because they did not fulfill the inclusion criteria, eight declined to participate, and 14 were unavailable. One participant was withdrawn from the study because of hypertension, which was discovered during the laboratory visit. The final sample comprised 64 participants, 30 in the control group (CG) and 34 in the e-sports group (E-SG).

Of the e-sports athletes, 19 competed in MOBA-LOL, 6 in FPS -Valorant, 5 in FPS-R6, and 4 in Battle Royale- FF. Twenty-four control participants reported playing e-sports occasionally or regularly during their leisure time, while four reported no e-sports participation, and two did not respond. Control participants reported playing a median of 10.0 h/week (range 0–35 h/week), whereas professional e-sports athletes reported a median of 55.0 h/week of gaming (range 30 to 105 h/week), indicating very limited overlap in gaming exposure between the groups.

All volunteers were male and the groups were well matched for age. Body weight, BMI, fat-free mass, absolute and relative body fat, and blood test results did not differ between the groups. However, sedentary time was markedly higher among e-sports athletes 12.09 (IQR: 2.54) vs. 7.74 (IQR: 3.20), *p* < 0.001 (Table 1 and Supplementary Table S1).

**Table 1:** Sociodemographic and clinical characteristics of the study sample.

Variable	CG (*n* = 30)	E-SG (*n* = 34)	*p*-value
Age (years)	21.9 ± 2.36	21.9 ± 2.63	0.902
Body weight (kg)	80.46 ± 14.04	79.60 ± 18.26	0.952
Body mass index (kg/m^2^)	26.31 ± 3.93	25.09 ± 5.24	0.381
Fat-free mass (kg)	66.16 ± 7.59	63.25 ± 11.8	0.300
Body fat (kg)	15.73 ± 9.92	15.29 ± 8.61	0.962
Body fat (%)	17.19 ± 9.53	17.82 ± 7.04	0.671
Sedentary time (h)	7.74; 3.20^#^	12.09; 2.54^#^	**<0.001**
Weekly gaming (h)	11.4 ± 9.66	56.9 ± 17.6	**<0.001**
Hemoglobin (g/dL)	15.27 ± 1.09	15.18 ± 0.96	0.735
PSQI (score)	6.14 ± 2.92	6.68 ± 2.80	0.445
Sleep quality (*n*; %)			0.490
Good (PSQI ≤ 5)	10 (34.5)	9 (26.5)	
Poor (PSQI > 5)	19 (65.5)	25 (73.5)	
Schooling level (*n*; %)			**<0.001**
Complete technical/vocational or college education	3 (10%)	0	
Ongoing technical/vocational or college education	27 (90%)	6 (17.6%)	
Stopped at elementary or high school	0	27 (82.4%)	
Family income (*n*; %)			0.226
Up to 10 minimum wages	16 (53.3)	13 (38.2)	
More than 10 minimum wages	14 (46.7)	21 (61.58)	
Known disease (*n*; %)			0.966
No	24 (82.8)	28 (82.4)	
Yes	5 (17.2)	6 (17.6)	
Tobacco smoking in the last 30 days (*n*; %)			0.192
No	26 (86.7)	25 (73.5)	
Yes	4 (13.3)	9 (26.5)	
Electronic cigarette uses in the last 30 days (*n*; %)			0.284
No	24 (80.0)	23 (67.6)	
Yes	6 (20.0)	11 (32.4)	
Cannabis use in the last 30 days (*n*; %)			0.917
No	25 (83.3)	28 (82.4)	
Yes	5 (16.7)	6 (17.6)	
Alcohol consumption in the last 30 days (*n*; %)			0.325
No	8 (26.7)	13 (38.2)	
Yes	22 (73.3)	21 (61.8)	
PHQ9 (*n*; %)			0.581
Mild depression	8 (26.7)	13 (38.2)	
Moderate depression	4 (13.3)	3 (8.8)	
Minimal depression	18 (60.0)	18 (52.9)	
HADS Anxiety (*n*; %)			0.128
Borderline	5 (16.7)	4 (11.8)	
Normal	22 (73.3)	30 (88.2)	
Positive screening	3 (10.0)	0 (0.00)	
HADS depression (*n*; %)			0.187
Borderline	1 (3.3)	6 (17.6)	
Normal	28 (93.3)	27 (79.4)	
Positive screening	1 (3.3)	1 (2.9)	
EQ5D dimensions (*n*; %)			
Mobility			0.482
Level 1	28 (93.3)	33 (97.1)	
Level 2	2 (6.7)	1 (2.9)	
Self-care			1.000
Level 1	30 (100.0)	34 (100.0)	
Usual activities			**0.028**
Level 1	26 (86.7)	34 (100.0)	
Level 2	4 (13.3)	0 (0.0)	
Pain			0.297
Level 1	26 (86.7)	26 (76.5)	
Level 2	4 (13.3)	8 (23.5)	
Anxiety and depression			0.619
Level 1	22 (73.3)	23 (68.6)	
Level 2	8 (26.7)	11 (31.4)	
Overall health score	79.6 ± 10.6	69.8 ± 20.0	**0.019**

Data are presented as mean ± standard deviation, median (IQR)^#^, or absolute frequency (*n*) and percentage (%). Group comparisons were performed using the student *t* test, chi-square test (*χ*^2^) or Fisher's exact test when appropriate. Variables included educational level, family income, presence of known diseases, smoking, electronic cigarette use, cannabis use, alcohol consumption, PHQ-9 depression categories, HADS anxiety and depression classifications, and EQ-5D dimensions (mobility, self-care, usual activities, pain, anxiety/depression), as well as sleep-quality categories. PSQI, Pittsburgh sleep quality index; PHQ-9, patient health questionnaire-9; HADS, hospital anxiety and depression scale.

Bold values represent significant differences between groups.

E-SG participants predominantly held high school degrees, which differed from the university students in the control group (*χ*^2^ = 25.1, *p* < 0.001). In contrast, family income did not differ between groups (*χ*^2^ = 1.47, *p* = 0.226), nor did the prevalence of self-reported diseases (*χ*^2^ = 0.001, *p* = 0.966). Tobacco smoking (*χ*^2^ = 1.70, *p* = 0.192) and vaping (*χ*^2^ = 1.25, *p* = 0.264) were numerically more frequent in the E-SG, but the difference did not reach statistical significance.

Participants in both groups reported similar sleep quality (*χ*^2^ = 4.77, *p* = 0.490), while screening for depression and anxiety also yielded similar results. Across the EQ-5D dimensions, most participants reported no problems (Level 1), although Level 2 responses were observed for mobility, usual activities, pain/discomfort, and anxiety/depression; all participants reported Level 1 for self-care. However, the E-SG assigned lower scores for their overall health status than the CG (69.8 ± 20.0 vs. 79.6 ± 10.6, *p* = 0.019) despite reporting fewer limitations for usual activities (*χ*^2^ = 4.84, *p* = 0.028) (Table 1).

### 3.1 Primary outcomes: physical and cognitive performance

Participants in the CG demonstrated superior handgrip strength (47.23 ± 8.69 kgf vs. 42.8 ± 8.88 kgf; *p* = 0.027) and cardiorespiratory fitness compared with the E-SG, with both percent predicted V̇O_2_peak (V̇O_2_peak%) and V̇O_2_ at the anaerobic threshold (V̇O_2_AT%) being significantly higher in the CG (106.7 ± 13.49 vs. 86.4 ± 12.05; *p* < 0.001 and 86.00 ± 13.35 vs. 68.5 ± 13.56; *p* < 0.001, respectively) (Figure 1, Supplementary Table S2). As for the cognitive tests, the E-SG participants consistently displayed faster working memory reaction time (*p* = 0.016), but performance in simple reaction time, choice reaction time, and episodic and verbal memory accuracy were otherwise similar between groups (Figure 2, Supplementary Table S2).

**Figure 1:**
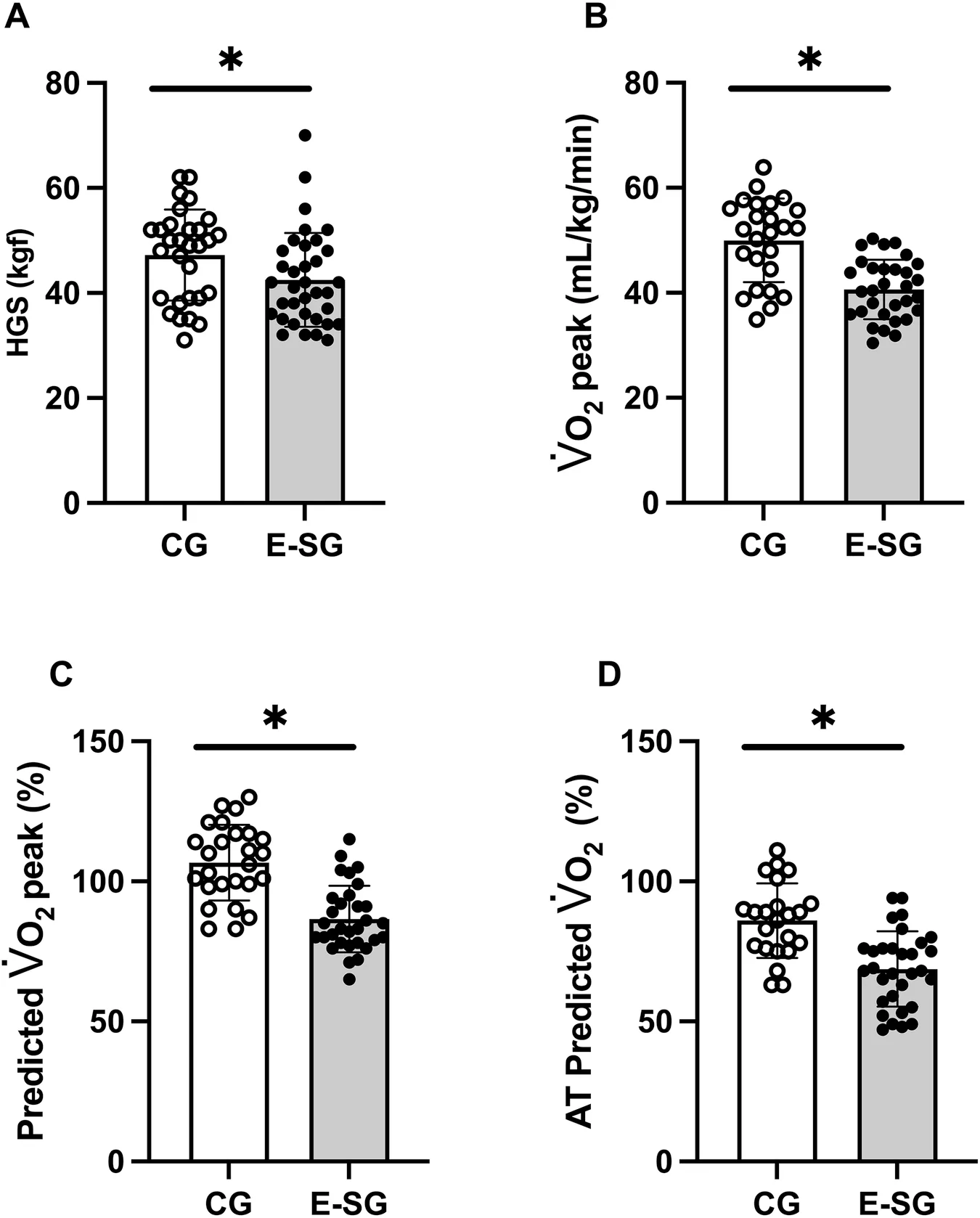
Comparison of muscle strength and cardiorespiratory fitness parameters between the control group (CG) and the e-sports group (E-SG). Panel **(A)** shows handgrip strength (HGS) expressed in kilogram-force (kgf). Panel **(B)** presents peak oxygen uptake (V̇O_2_peak) in millimeters per kilogram per minute (mL/kg/min). Panel **(C)** displays predicted V̇O_2_peak expressed as a percentage of age- and sex-specific reference values, and panel **(D)** illustrates oxygen uptake the anaerobic threshold (AT), also expressed as a percentage (%) of predicted values. Individual data points are plotted over the bars, which represent group means and standard deviation. *Statistically significant difference between groups.

**Figure 2:**
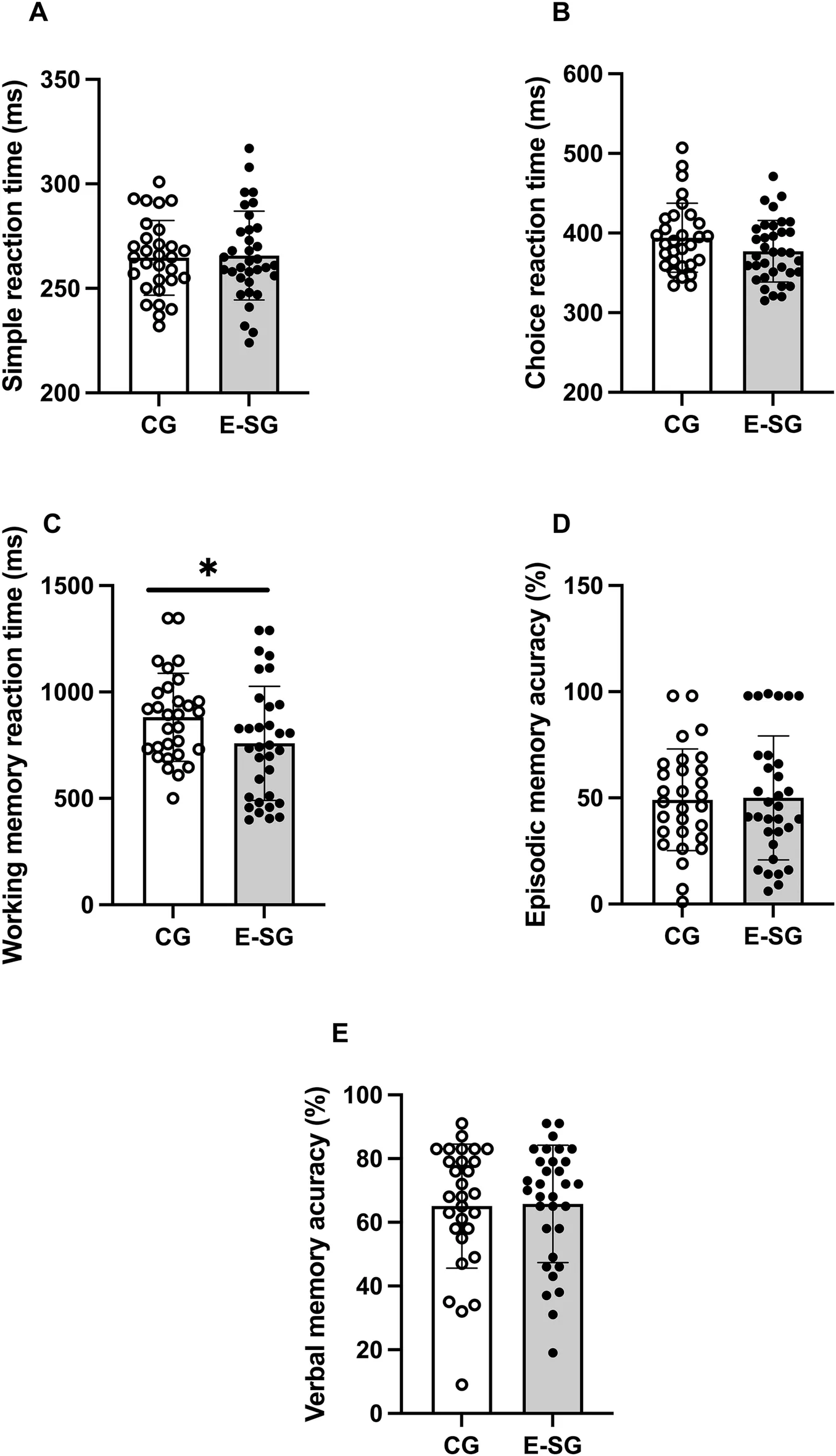
Cognitive performance outcomes in the control group (CG) and the e-sports group (E-SG). Panel **(A)** presents simple reaction time, panel **(B)** shows choice reaction time, and panel **(C)** displays working memory reaction time, all expressed in milliseconds (ms). Panels **(D)** and **(E)** illustrate episodic memory accuracy and verbal memory accuracy, respectively, both expressed as percentages (%). Individual data points are overlaid on the bars, which represent group means and standard deviation. *Statistically significant difference between groups.

### 3.2 Determinants of physical and cognitive performance and their impact on professional performance

Multivariate linear regression models were used to identify independent predictors for handgrip strength and aerobic capacity assessed by both V̇O_2_peak% and V̇O_2_AT%. The model for handgrip strength demonstrated only modest explanatory capacity (*R*^2^ = 0.246, adjusted *R*^2^ = 0.208) (Supplementary Table S3). In contrast, the model for V̇O_2_peak% showed robust explanatory capacity (*F* = 11.7 (41,5); *R*^2^ = 0.59, adjusted *R*^2^ = 0.53; *p* < 0.001), with two identifiable independent predictors: fat-free mass (*p* = 0.007) and group (*p* < 0.001), with E-SG participants demonstrating markedly lower V̇O_2_peak% (*p* < 0.001). For V̇O_2_AT%, the model demonstrated moderate explanatory strength (*F* = 6.94 (38,5); *R*^2^ = 0.47, adjusted *R*^2^ = 0.41; *p* < 0.001). Sedentary time was inversely associated with V̇O_2_AT% (*β* = −1.38, *p* = 0.035), such that each additional hour spent sitting per weekday predicted an estimated 1.4-percentage-point decrement in V̇O_2_%AT. Group classification was also a significant independent determinant, with participants in the E-SG exhibiting lower V̇O_2_AT% compared with those in the CG (*β* = −10.33, *p* = 0.028), corresponding to an adjusted deficit of approximately ten percentage points. All remaining predictors were not significant in the multivariate context (Table 2).

**Table 2:** Multivariate linear regression models for aerobic capacity parameters (*n* = 64).

Outcomes						
Variables	V̇O_2_peak (% of predicted)			V̇O_2_ (% of predicted AT)		
	Mean (*β*)	95% CI	*p*-value	Mean (*β*)	95% CI	*p*-value
CG vs. E-SG	−14.7	−22.85; −6.53	**<0.001**	−10.33	−19.47; −1.18	**0.028**
Sedentary time during week (h)	−1.05	−2.20; 0.10	0.073	−1.38	−2.66; −0.106	**0.035**
Hemoglobin (g/dL)	2.80	−0.49; 6.09	0.098	3.31	−0.41; 7.04	0.080
Fat-free mass (kg)	0.49	0.14; 0.84	**0.007**	0.30	−0.07; 0.68	0.116
Age (years)	0.22	−1.23; 1.68	0.761	0.22	−1.44; 1.89	0.786

Multivariate linear regression model examining independent predictors of absolute and predicted oxygen uptake (V̇O_2_peak). Estimates adjusted for age, hemoglobin concentration, sedentary time, and fat-free mass. Statistical significance set at *p* ≤ 0.05. Model fit: *R*^2^ and AIC. For aerobic fitness outcomes, group classification (CG vs. E-SG), age, hemoglobin concentration, sedentary time during the week, and fat-free mass were included as covariates. %, percent; AT, anaerobic threshold; B, Beta; CI, confidence interval; CG, control group; E-SG, e-sports group; h, hours; g, grams; dL, deciliter; kg, kilograms.

Bold values represent significant differences between groups.

E-sports athletes showed significantly faster working memory than control subjects (736 ms vs. 893 ms; Supplementary Table S2). In the univariate analyses, group classification was significantly associated with working memory performance (*β* = −151.0, 95% CI −268.0 to −32.8, *p* = 0.013; *R*^2^ = 0.09). Schooling level was also significantly associated with the outcome, with participants with elementary or high school education showing faster working memory than those with technical/vocational or college education (*β* = −124.0, 95% CI −244.0 to −4.29, *p* = 0.043; *R*^2^ = 0.06). Training time was inversely associated with working memory performance, so that longer gaming hours translated into faster working memory (*β* = −2.98 per hour/week, 95% CI −5.32 to −0.644, *p* = 0.013; *R*^2^ = 0.10). PSQI score, anxiety categories, depression categories, and family income were not significantly associated with the outcome in the univariate analyses.

In Model 1, adjusted for PSQI score and anxiety and depression symptom categories, group classification remained significantly associated with working memory performance (*β* = −165.36, 95% CI −296.55 to −34.2, *p* = 0.014) while others were not independently associated with the outcome. Model 1 explained 13.4% of the variance in working memory performance (*R*^2^ = 0.134). After the addition of family income in Model 2, the association with group classification remained significant and slightly increased in magnitude (*β* = −178.3, 95% CI −310.59 to −46.0, *p* = 0.009), with the proportion of explained variance increasing modestly to 15.9% (*R*^2^ = 0.159). In Model 3, the schooling level was additionally included. After this adjustment, the coefficient for group classification was attenuated (*β* = −123.4, 95% CI −278.85 to 32.0) and was no longer statistically significant. The proportion of explained variance increased modestly to 18.7% (*R*^2^ = 0.187) (Supplementary Table S4).

The impact of both physical and cognitive performances on championship ranking was investigated using further regression models. Ultimately, in the univariate analyses, working memory showed a small but positive association with rank percentile (*β* = 0.05, 95% CI 0.01 to 0.09; *p* = 0.013). In the multivariable regression analyses, adjustment for potential confounders in Models 1 and 2 attenuated most associations, with no statistically significant effects observed for training time, PSQI, or reaction time measures. In the final adjusted model (Model 3), training time emerged as a borderline predictor of rank percentile (*β* = 0.70, 95% CI −9.11 × 10^4^ to 1.40; *p* = 0.050), indicating a tendency toward higher performance with greater weekly training volume. Working memory remained independently associated with rank percentile in the final model (*β* = 0.076, 95% CI 0.01 to 0.13; *p* = 0.011), suggesting that faster working memory responses were associated with better performance outcomes (Table 3).

**Table 3:** Linear regression analyses examining the association between training, sleep, and cognitive performance variables and competitive performance percentile in e-sports players (*n* = 34).

Variables	Univariate analysis			Model 1			Model 2			Model 3		
	*β*	95% CI	*p*-value	*β*	95% CI	*p*-value	*β*	95% CI	*p*-value	*β*	95% CI	*p*-value
Training time (hours/week)	0.51	−0.08 to 1.11	0.090	0.43	−0.23 to 1.1	0.19	0.43	−0.25 to 1.11	0.203	0.70	−9.11 × 10^4^ to 1.40	**0.050**
PSQI	2.21	−1.45 to 5.87	0.226	1.11	−2.88 to 5.1	0.572	1.14	−2.95 to 5.23	0.571	−1.19	−5.46 to 3.07	0.567
Simple reaction (ms)	0.270	−0.23 to 0.77	0.282	0.25	−0.25 to 0.75	0.309	0.24	−0.27 to 0.76	0.339	−0.05	−0.61 to 0.49	0.832
Choice reaction time (ms)	4.31 × 10^−4^	−0.28 to 0.28	0.998	—	—	—	0.02	−0.29 to 0.34	0.858	−0.12	−0.50 to 0.25	0.494
Working memory (ms)	0.05	0.01 to 0.09	**0.013**	—	—	—	—	—	—	0.076	0.01 to 0.13	**0.011**

Univariate and multivariate linear regression models evaluating the associations between training characteristics, sleep quality, and cognitive performance measures with players' competitive performance percentile. The outcome represents the percentile ranking achieved by players in official competitions, including the South America League 2025 Stage 1, Champions Tour 2025 Americas Kickoff, CBLOL Academy Split 1 2024, and CBLOL Split 1 2024. Models 1 and 2 correspond to progressively adjusted regression models, while Model 3 represents the final fully adjusted (for training time, PSQI, simple reaction time, choice reaction time, and working memory) model. Regression coefficients (*β*), 95% confidence intervals (95% CI), and *p*-values were reported. Statistical significance was set at *P* < 0.05. PSQI, Pittsburgh sleep quality index; ms: milliseconds.

Bold values represent significant differences between groups.

## 4 Discussion

This study provides a comprehensive characterization of the physical conditioning, cognitive performance, mental health, and quality of life of professional e-sports athletes while examining how these domains relate to their performance in championships. Our findings indicate that e-sports athletes showed faster working memory performance in the primary regression model, which was associated with better performance in championships. In contrast, e-sports athletes also showed significantly higher sedentary time and markedly lower cardiorespiratory conditioning and muscle strength than age- and sex-matched university students. This highlights the complex interplay between professional needs and their potential long-term effects on general health.

The choice to compare e-sports athletes with university students was not random. First, most of our e-sports athletes were at an age where they would have been expected to attend college had they not chosen to become professional players. Second, university students are also expected to show marked sedentary behavior driven by long hours spent sitting in class or studying at home. Third, higher education is predicted to result in better cognitive performance. Therefore, comparing these two groups would allow us to understand the real impact of the routine adopted by e-sports athletes on what would have been expected of them otherwise.

Working memory plays a central role in competitive gaming by supporting rapid decision-making, task switching, and real-time updating of information (17, 18). While univariate analysis showed both higher educational level and professional e-sports playing to be associated with faster working memory, the fact that these associations did not remain significant on multivariate analysis weakened the finding and prevented any definite conclusions to be drawn. However, it is noteworthy that e-sports group showed on average faster working memory than the control group despite their lower educational level, especially as working memory was found to be associated with performance in e-sports championships. Additionally, there was a trend towards better professional performance with longer training hours, which raises the hypothesis that practicing electronic sports could perhaps improve working memory. The absence of associations between working memory and sleep quality, mental health variables or substance use further supports this hypothesis, which warrants further investigation.

On the other hand, we did not find significant differences in simple reaction time, choice reaction time, episodic memory, or verbal memory between e-sports athletes and the control group. There are several possible explanations for this, with the first being that the cognitive advantage of game training might be selective rather than global. Videogame playing has been shown to improve enumeration and multiple object tracking ability, perhaps through changes in visual short-term memory skills (19), but the neurocognitive tests that we applied did not exactly resemble the scenario the athletes are used to, with verbal memory tests showing words rather than objects. In addition, it is important to stress that the comparator group was composed of college students, who themselves were expected to present with higher-than-average neurocognitive skills. Finally, one alternative hypothesis is that poor sleep hygiene and habits could be offsetting the positive impacts of game practice on reaction time. One of the determinants of attention is sleep quality, and interestingly, our study showed that most participants in both groups reported poor overall sleep quality. Indeed, there have been studies showing that improving sleep quality can lower reaction time in dedicated tests among e-sports players (20).

One of the most relevant findings of this study was the markedly reduced physical fitness observed among e-sports athletes. Aerobic capacity can be affected by a number of factors, including body composition, comorbidities, and lifestyle (including smoking and exercise) (21). Notably, in our study, we evaluated only individuals who were considered generally healthy, and no differences were noted in hemoglobin concentrations, lung function, or tobacco consumption between the groups. Therefore, we hypothesized that this reduced physical fitness was driven mainly by body composition and sedentary time, as these two variables were significantly associated with VO_2_ at the AT and at peak exercise. Our findings are in line with previous studies showing that e-sports players tend to have reduced fat-free mass, physical activity, and aerobic capacity (22, 23). However, the concept of the unfit e-sports player has been recently challenged by Ketelhut et al. (24), who found that e-sports players had a similar aerobic capacity to non-players. Still, it must be stressed that their sample was predominantly composed of amateur players who practiced on average 2 h a day, while we studied only professional e-sports athletes who practiced on average 8.1 h/day, with consequently high sedentary time by current clinical standards (25).

Increased sedentary time has been shown to elevate cardiovascular risk (25, 26) and has been repeatedly associated with poor quality of life (27, 28). While at this early competitive stage we did not find any metabolic abnormalities associated with prolonged inactivity and deconditioning (e.g., diabetes or dyslipidemia) our e-sports athletes already showed lower perceived quality of life. Interestingly, while athletes reported fewer limitations in usual activities on the EQ-5D than control subjects and showed similar prevalence of anxiety and depression, their overall health state scores were lower than those of controls. This apparent paradox may reflect preserved functional capacity for non-physically demanding daily tasks, alongside a heightened awareness of compromised health, potentially related to fatigue, physical discomfort, or concerns regarding long-term health consequences of their adopted lifestyle.

## 5 Practical implications

In this setting, routine exercise training can be a necessary intervention for professional e-sports players to counterbalance some of the deleterious effects of long hours of gaming on their health, although this study did not directly evaluate the effects of such intervention. Adopting an exercise routine is key to improving cardiovascular health and aerobic capacity (29), as well as reducing chronic conditions associated with prolonged inactivity, such as neck and low back pain (30). In addition, exercise is known to assist in quitting tobacco use (31) and improving mental health and sleep quality (32, 33), both of which can impact attention, response time, and working memory. Indeed, a short-term exercise intervention was shown to improve key cognitive and motor skills among FPS e-sports players (23). While its actual impact on professional performance is likely less significant than game training itself, athletes' health and quality of life should be considered paramount, and exercise might play a role in reducing burnout and other health conditions that so often make players drop out of professional e-sports at still an early age (34).

## 6 Limitation and strengths

Our study has several limitations. First, its cross-sectional design precludes causal inference and allows only the identification of associations between professional e-sports playing, cognitive performance, and physical conditioning. Second, the recruitment strategy for control participants, which relied on electronic communications at university campuses, may have introduced selection bias, as students with greater interest in exercise or electronic gaming may have been more likely to participate. Third, the all-male sample does not allow generalization of the findings to female professional e-sports players. Fourth, the relatively small sample size is an important limitation, particularly given the broad range of outcomes assessed, which could have introduced both Type I and Type II errors. The small sample size also precluded a more in-depth analysis of the e-sports group according to the modalities played by participants.

One major limitation of the neurocognitive analysis was the choice of tests that we applied. The Ubrain® tests had not been previously validated in this population and our study did not seek to validate them. Even though a myriad of tests measuring core brain functions, such as attention, processing speed, visual learning, working memory, and task switching have been used to assess cognitive skills in e-sports players, there is no consensus or preference for either one. Specifically for computer-based tests like the ones we applied, reference values for the Brazilian population are lacking. For that reason, we did not analyze the results as percentiles or categories, but rather as absolute values to be compared between groups. Still, the markedly lower educational level of e-sports players compared to control subjects further complicated the interpretation of the neurocognitive findings by probably attenuating the differences encountered between the groups. However, this educational level imbalance was expected based on the average age of professional e-sports players worldwide.

Finally, the fact that electronic gaming was a common leisure activity among control subjects may have also reduced the between-group differences, especially in terms of the cognitive skills that can be trained by e-sports playing. Nevertheless, amateur playing of internet games is highly prevalent among university students in general, and particularly among those in the fields of health sciences, technology, and engineering. In that sense, our study sample is reflective of the real world, and we believe this increases its external validity.

Our study also has several strengths. First, it is one of the few studies dedicated to analyzing only professional-level e-sports athletes. Second, we used gold standard physiological assessments of physical performance and applied validated screening tools to characterize mental health, sleep, and QoL, allowing a comprehensive view of how these factors might interact. Third, we devised a metric of professional success to explore how physical and cognitive functions might impact e-sports athletes' performance in championships.

## 7 Conclusion

Professional e-sports athletes exhibited reduced aerobic fitness, lower fat-free mass, and higher sedentary behavior compared to matched university students. In addition, they demonstrated faster working memory despite, overall, having lower educational level. While cognitive efficiency and training time might be relevant to enhance competitive performance, physical fitness and metabolic markers did not directly influence outcomes during these early career stages. Our findings reinforce the cognitive demands of e-sports while highlighting clinically relevant health vulnerabilities, supporting future clinical trials to test the impact of physical training and preventive health strategies on sustainable performance and long-term athlete well-being.

## Data Availability

The raw data supporting the conclusions of this article will be made available by the authors, without undue reservation.
