# Supplementary material for: Aerobic capacity, strength, and cognitive performance of e-sports athletes and their determinants
Source: Front Sports Act Living. 2026 Sep 18;8:1940173. doi: 10.3389/fspor.2026.1940173 (PMC13630747; doi:10.3389/fspor.2026.1940173)
Supplement: Supplementary file 1 [file Datasheet1.pdf]

Physical and cognitive performance of e-sports athletes: the complex interplay between professional needs and their potential health impacts

Supplementary material

Below we included additional detailed information on each of the following topics:

1. INSTRUMENTS AND PROCEDURES

1.1 PARTICIPANTS, RECRUITMENT STRATEGIES, AND SAMPLE SIZE

Individuals with ongoing orthopedic or muscle injuries, uncontrolled hypertension or arrhythmias, as well as those with significant or uncontrolled cardiopulmonary or neuromuscular diseases that could potentially impair aerobic capacity or place the subject under increased risk were excluded.

The study was conducted on convenience sampling, which introduces limitations regarding external validity and generalizability[1], so data should be analyzed with caution.

No design violations occurred.

1.2 ANTHROPOMETRIC MEASUREMENT AND BODY COMPOSITION

Body mass and height were measured in triplicate using a digital scale and a wall-mounted stadiometer, respectively. Both measurements were taken with participants wearing light clothing. All measurements were performed individually in a private room by a trained evaluator (anthropometrist) to ensure participant comfort and privacy. Subsequently, in the same room, participants remained in the supine position resting for 10 minutes to allow for bioelectrical impedance analysis of body composition (BIA, **Biodynamics® model 450)**. All assessments were conducted after at least four hours of absolute fasting in the supine position. Participants were also instructed to avoid diuretics, caffeine intake, high-intensity physical exercise and excessive or restrictive fluid intake during the 24 hours preceding testing.

1.3 NEUROCOGNITIVE TESTS

The Ubrain® tests (https://ubrain.com.br) were based on the CogState Brief Battery® (https://www.cogstate.com/publications/tag/cogstate-brief-battery/), a quick computer-based test which has been used to measure core brain functions like attention, processing speed, visual learning, and working memory in a myriad of scenarios. The Ubrain® tests follow the exact same pattern of the CogState tests, with slight variations (for instance, instead of asking if a card is red or not, it asks if a target is red or not).

Neither the CogState Brief Battery® nor the Ubrain® tests have been validated for e-sports players and reference values for the Brazilian population are lacking. For that reason, we did not analyze the results as percentiles or categories, but rather as absolute values to be compared between groups. At the time we were designing the study, there had been very limited literature on cognitive testing of e-sports players, and no gold standard assessment exists until this date. On the other hand, The Ubrain® tests were quick computer-based tests, the instructions were provided in Portuguese, the platform had been previously used by the sports medicine doctor of one of the teams enrolled in our research to assess their players cognitive performance, and we were granted free access to the tests for this research. For all of these reasons, Ubrain® tests were our choice of cognitive assessment in this study.

Ubrain® tests generally involved showing an image on a computer screen (playing cards, words or a target, depending on the test) and asking participants to take some specific action using the keyboard or the computer mouse, assessing how fast and how accurate participants responded.

Volunteers underwent 5 standardized tests using the Ubrain® platform, in the following order: 1. Episodic Memory Test (EMT): 90 cards with geometric shapes were presented sequentially. Participants were asked to respond "yes" (pressing the letter J on the keyboard) if the card had appeared any time before or "no" (pressing the letter F on the keyboard) if it had not. A correct response index was calculated; 2. Simple Reaction Test (SRT): 30 green targets were presented sequentially on the screen. For each new target, the participant should press the "J" key as quickly as possible. The reaction time between the target appearance and the key press was recorded; 3. Choice Reaction Test (CRT): 30 blue or red targets were presented sequentially. If the target was red, the participant should press "J"; if blue, they should press "F." Both the accuracy index and reaction time for correct responses were recorded; 4. Verbal Memory Test (VMT): 90 words were presented sequentially. Participants needed to respond

"yes" if the word had appeared previously ("J" on the keyboard) or "no" if it had not ("F" on the keyboard). A correct response index was calculated; and 5. Working Memory Test (WMT): 40 cards were presented sequentially. Participants had to remember only the last card presented. They responded "yes" ("J" on the keyboard) if the new card matched the immediately previous one or "no" ("F" on the keyboard) if it did not. A correct response index was calculated.

Every test was preceded by a 30 second training session so that volunteers could be familiarized with the type of assessment beforehand. Memory and reaction time tests always followed the same order and we made sure the cognitive assessment was always performed after volunteers had already been offered a small meal and before they underwent cardiopulmonary exercise testing.

1.4 PHYSICAL FITNESS AND STRENGTH ASSESSMENT

Physical fitness was assessed using a cardiopulmonary exercise test (CPET) performed on a treadmill (Super ATL, Inbrasport, Porto Alegre, RS, Brazil). Following the BIA assessment, volunteers were given a light standardized meal (1 g of carbohydrate per kilogram of body mass)[2] and waited 60 -90 minutes to perform the CPET.

The incremental ramp protocol comprised 2 minutes of resting measurements, a 2-minute warm up and incremental exercise of 20 or 30 second stages until exhaustion, which was then followed by 2 minutes of active recovery and 2 minutes of passive recovery. The incremental phase consisted of progressive increases in speed and slope which were tailored to resemble a ramp increment and allow exercise to last 8 to 12 min.[3] Breath-by-breath gas exchange was measured using a metabolic system (K5, COSMED, Rome, Italy) along with beat-by-beat heart rate monitoring (GARMIN® chest strap). Peripheral oxygen saturation was assessed using a pulse oximeter (NONIN Onyx Vantage®). The modified Borg scale (0–10)[4] was applied every two minutes to assess perceived breathlessness and leg discomfort.

Tests were considered maximal if at least two of the following criteria were met: (i) heart rate greater than or equal to 85% of the age-predicted maximum according to the equation proposed by Tanaka et al;[5] (ii) modified Borg score for leg fatigue or dyspnea greater than 5; (iii) respiratory exchange ratio ($\dot{V}CO_2/\dot{V}O_2$) ≥ 1.10. If a maximal test was obtained, peak oxygen consumption (peak $\dot{V}O_2$) was evaluated as a measure of aerobic capacity, being recorded both

in absolute values and percent of the predicted value for sedentary, healthy individuals of the same sex, age, and weight, according to the predictive equations of Neder et al.[6]

Additionally, handgrip strength was assessed using a hydraulic hand dynamometer (JAMAR®) and compared with predicted values.[7] The strength assessment followed the standardized procedure recommended by the American Society of Hand Therapists.[8] Participants performed the handgrip strength tests with both hands (dominant and non-dominant). They were instructed to perform three maximal contractions of three seconds each per hand, with a 30-second rest period between contractions. For data analysis, the highest obtained value was recorded.

1.5 PROFESSIONAL PERFORMANCE

Publicly available championship results:

https://oracleselixir.com;

https://Vlr.gg;

https://liquipedia.net/rainbowsix/Six_Invitational/2025

1.6 BIOCHEMICAL ANALYSIS

For the blood cell count and glycated hemoglobin, whole blood was analyzed. Serum samples were analyzed for iron, ferritin, thyroid-stimulating hormone (TSH), thyroxine (T4), vitamin B12, and vitamin D assessment. Serum samples were analyzed for iron, ferritin, thyroid-stimulating hormone (TSH), thyroxine (T4), vitamin B12, and vitamin D assessment. All analyses were performed at São Camilo Hospital, following their standard procedures. For all participants, blood tests were the last procedure of the visit, which happened at the end of a morning or afternoon period, according to the participants availability.

1.7 STATISTICAL ANALYSIS

All statistical analyses were performed using Jamovi software (version 2.6, The Jamovi Project, Sydney, Australia). Statistical significance was set at $p \leq 0.05$ for two-sided tests. Continuous variables were assessed for normality using the Shapiro–Wilk test and were expressed as mean and standard deviation or median and interquartile range, as appropriate. Between-group comparisons were performed using independent-samples t-tests or Welch's t-tests when homogeneity of variances was violated, and Mann–Whitney U tests for non-normally distributed variables. Categorical variables were expressed as absolute and relative frequencies and compared using chi-square or Fisher's exact tests, as appropriate.

No formal a priori sample size or power calculation was performed. This study was designed as an exploratory cross-sectional investigation of professional e-sports athletes, and recruitment was primarily determined by the availability of eligible professional players and the feasibility of completing the extensive physiological, neurocognitive, and questionnaire-based assessment protocol. Accordingly, the regression analyses should be considered exploratory and were intended to identify potential associations rather than to provide confirmatory estimates of independent effects.

Multivariable linear regression models were fitted for handgrip strength, $\dot{V}O_2$peak ($mL \cdot kg^{-1} \cdot min^{-1}$), percentage of predicted $\dot{V}O_2$peak, percentage of predicted $\dot{V}O_2$peak at the anaerobic threshold, and working memory performance. Candidate predictors were defined a priori based on biological plausibility, empirical evidence, and potential role as confounding factors. Automated or exclusively data-driven variable selection procedures were not used. Given the sample size, the model construction also considered parsimony to avoid excessive parameterization, particularly for categorical covariates requiring multiple coefficients.

For working memory reaction time, the primary model included group classification, sleep quality, HADS anxiety and depression categories, and family income. Because educational level differed between groups and may influence cognitive performance, a second model additionally included educational level. These sequential models were used to assess the robustness of the association between professional e-sports status and working memory performance while considering the trade-off between confounding control and model complexity. Model fit was evaluated using R^2, adjusted R^2, and the Akaike Information Criterion (AIC), together with the incremental variance explained by additional covariates. Regression results are presented as unstandardized regression coefficients (B), 95% confidence intervals (95% CI), and corresponding p values. Regression assumptions, including linearity,

homoscedasticity, normality, and independence of residuals, and multicollinearity, were assessed as detailed in the Supplementary Material.

No formal adjustment for multiple comparisons was applied because the study was exploratory and evaluated prespecified outcomes across distinct physical fitness, neurocognitive, mental health, behavioral, and quality of life domains rather than a single confirmatory family of hypotheses. Accordingly, individual p-values should be interpreted as exploratory and in conjunction with effect estimates, 95% confidence intervals, and consistency across analyses.

2. RESULTS

Table S1: Blood test results of study volunteers (n = 60).

Variable	CG	E-SG
Vitamin D (ng/mL)	26.9 ± 7.5	21.6 ± 5.8
Vitamin B12 (pg/mL)	581.1 ± 146.3	568.5 ± 224.4
Iron (µg/dL)	104.3 ± 37.9	111.2 ± 29.3
Ferritin (ng/mL)	230.2 ± 109.9	219.5 ± 103.1
TSH (µIU/mL)	2.2 ± 1.8	2.3 ± 1.1
Free T4 (ng/dL)	1.3 ± 0.1	1.4 ± 0.2
HbA1C (%)	5.2 ± 0.4	5.2 ± 0.3

Legend: Data are presented as means ± standard deviation. CG, control group; E-SG, e-sports group; ng, nanogram; mL, milliliter; pg, picogram; µg, microgram; dL, deciliter; IU, international units; %, percent; TSH, Thyroid Stimulating Hormone; T4, thyroxine; HbA1C, Glycated hemoglobin.

Table S2. Physical and cognitive performance of study volunteers (n = 64).

Variable	Groups	Median	P25; P75	p-value
HGS (kgf)	CG	49.50	39.0; 52.0	0.027
	E-SG	41.50	36.0; 48.0	
$\dot{V}O_2$peak (ml/kg/min)	CG	52.10	44.50; 56.0	**<0.001**
	E-SG	40.85	36.45; 44.65	
$\dot{V}O_2$peak (% of predicted)	CG	108.0	99.0; 116.5	**<0.001**
	E-SG	83.0	78.25; 93.50	
$\dot{V}O_2$ @ AT (% of predicted)	CG	88.0	76.5; 91.5	**< 0.001**

	E-SG	68.0	57.5; 76.0	
Heart rate peak (bpm)	CG	193.0	187.0; 195.0	0.194
	E-SG	188.0	184.25; 196.5	
RER peak	CG	1.13	1.11; 1.21	0.023
	E-SG	1.19	1.16; 1.23	
$\dot{V}_E$ peak / MVV	CG	0.76	0.69; 0.82	0.072
	E-SG	0.67	0.64; 0.77	
$\dot{V}_E$ / $\dot{V}CO_2$ @ AT	CG	26.0	22.7; 30.1	0.469
	E-SG	27.8	25.58; 29.97	
Borg scale - leg fatigue	CG	7.0	5.0; 8.5	0.535
	E-SG	7.0	5.0; 9.0	
Borg scale - dyspnea	CG	7.0	5.0; 9.0	0.640
	E-SG	8.0	5.75; 9.0	
Simple reaction time (ms)	CG	265.0	254.0; 273.25	0.825
	E-SG	261.0	255.0; 277.0	
Choice reaction time (ms)	CG	388.0	361.25; 416.5	0.099
	E-SG	373.0	350.25; 401.0	
Working memory time (ms)	CG	893.5	730.7; 986.0	**0.016**
	E-SG	736.0	493.5; 838.0	
Episodic memory accuracy	CG	48.0	34.0; 63.0	0.897
	E-SG	46.0	31.0; 68.0	
Verbal memory accuracy	CG	68.0	58.0; 79.0	0.889
	E-SG	72.0	53.5; 79.0	

Legend: Data are expressed as the median and the 25th and 75th percentiles. Group comparisons were conducted using the independent-samples Student's t-test for normally distributed variables and the Mann–Whitney U test for nonparametric variables. HSG, handgrip strength; $\dot{V}O_2$peak, peak oxygen uptake; AT, anaerobic threshold; RER, respiratory exchange ratio peak; $\dot{V}_E$, ventilation; MVV, maximal voluntary ventilation; / $\dot{V}CO_2$, carbon dioxide production; bpm, beats per minute; ms, milliseconds. Significant values are indicated by $p < 0.05$.

Table S3. Multivariate Linear Regression Model for Handgrip Strength (n = 64).

Outcomes			
**Variables**	**HGS (kgf)**		
	Mean (β)	95% CI	p-value
CG vs. E-SG	-3.31	-7.37; 0.746	0.108
Sedentary time during week (h)	-	-	-
Hemoglobin (g/dL)	-	-	-
Lean body mass (kg)	0.38	0.18; 0.59	**<0.001**

Age (years)	0.03	-0.78; 0.84	0.939

Legend: Multivariate linear regression model examining independent predictors of maximal handgrip strength (adjusted for age and fat-free mass). Estimates adjusted for age, hemoglobin concentration, sedentary time, and fat-free mass. Model fit: R^2 and AIC. For handgrip strength, the model included group classification, age, and lean body mass. Regression coefficients (β), 95% confidence intervals (95% CI), and p values are reported. Statistical significance was set at $p < 0.05$. HGS, handgrip strength; CG, control group; E-SG, e-sports group; h, hours; g, grams; dL, deciliter; kg, kilogram.

Table S4. Multivariate Linear Regression Model for Working Memory (n = 64).

Variables	**Univariate analysis**			**Model 1 (R^2 = 0.134)**			**Model 2 (R^2 = 0.159)**			**Model 3 (R^2 = 0.187)**		
	β	95% CI	p-value; R^2	β	95% CI	p-value	β	95% CI	p-value	β	95% CI	p-value
CG vs. E-SG	-151.0	-268.0 - -32.8	**0.013; 0.09**	-165.36	-296.55 - -34.2	**0.014**	-178.3	-310.59 - -46.0	**0.009**	-123.4	-278.85 – 32.0	**0.117**
PSQI (score)	13.3	-8.82 – 35.3	0.234; 0.02	17.87	-6.15 – 41.9	0.142	20.7	-3.64 – 45.1	0.094	24.4	-0.442 – 49.2	**0.054**
HADS (Anxiety)												
Borderline vs Normal	-12.1	-189 – 165	0.892; 0.00	-43.13	-221.26 – 135.0	0.629	-63.9	-244.4 - 116.7	0.481	-70.9	-250.57 – 108.7	0.432
Positive Screening vs. Normal	31.6	-259 – 322	0.828; 0.00	-108.7	-410.7 - 193.2	0.473	-140.4	-445.46 – 164.6	0.360	-182.7	-492.28 – 127.0	0.242
HADS (Depression)												
Borderline vs Normal	48.4	-148 – 245	0.623; 0.00	9.90	-189.9 – 209.8	0.921	-25.8	-233.17 – 181.5	0.804	-19.1	-225.29 – 187.1	**0.853**
Positive Screening vs. Normal	96.1	-295 – 487	0.625; 0.00	-20.42	-387.0 – 346.2	0.911	-27.2	-392.38 – 338.0	0.882	-96.1	-473.52 – 281.3	**0.611**
Family Income												
Up to 10 minimum wages vs. > 10 minimum wages	14.6	-110 – 139	0.816; 0.00	-	-	-	82.6	-52.19 – 217.5	0.224	109.7	-30.31 – 249.8	**0.122**
Schooling Level												
Elementary and high school vs. technical/vocational and college education	-124	-244 - -4.29	**0.043; 0.06**	-	-	-	-	-	-	-110.4	-277.44 – 56.6	**0.190**
Training time (hours/week)	-2.98	-5.32 - -0.644	**0.013; 0.10**	-	-	-	-	-	-	-	-	-

Legend: Multivariable linear regression models assessing factors associated with working memory performance (n = 64). Model 1 included group classification, sleep quality assessed by the Pittsburgh Sleep Quality Index (PSQI), and anxiety and depression symptom categories assessed by the Hospital Anxiety and Depression Scale (HADS). Model 2 additionally included family income, and Model 3 additionally included schooling level. Weekly gaming/training exposure was evaluated in the univariate analysis but was not included in the multivariable models because of its close relationship with group classification, in order to reduce collinearity and model redundancy (VIF = 2.05; tolerance = 0.489). Regression coefficients (β), 95% confidence intervals (95% CI), p values, and coefficients of determination (R^2) are reported. Statistical significance was set at $p < 0.05$. E-SG, e-sports group; CG, control group; PSQI, Pittsburgh Sleep Quality Index; HADS, Hospital Anxiety and Depression Scale; VIF, variance inflation factor.

REFERENCES

1. Andrade C. The Inconvenient Truth About Convenience and Purposive Samples. *Indian J Psychol Med* 2021;43(1):86–88. doi: 10.1177/0253717620977000 [published Online First: 20201217]
2. Thomas DT, Erdman KA, Burke LM. American College of Sports Medicine Joint Position Statement. Nutrition and Athletic Performance. *Med Sci Sports Exerc* 2016;48(3):543–68. doi: 10.1249/MSS.0000000000000852
3. Buchfuhrer MJ, Hansen JE, Robinson TE, et al. Optimizing the exercise protocol for cardiopulmonary assessment. *J Appl Physiol Respir Environ Exerc Physiol* 1983;55(5):1558–64. doi: 10.1152/jappl.1983.55.5.1558
4. Borg GA. Psychophysical bases of perceived exertion. *Med Sci Sports Exerc* 1982;14(5):377–81.
5. Tanaka H, Monahan KD, Seals DR. Age-predicted maximal heart rate revisited. *J Am Coll Cardiol* 2001;37(1):153–6. doi: 10.1016/s0735-1097(00)01054-8
6. Neder JA, Nery LE, Peres C, et al. Reference values for dynamic responses to incremental cycle ergometry in males and females aged 20 to 80. *Am J Respir Crit Care Med* 2001;164(8 Pt 1):1481–6. doi: 10.1164/ajrccm.164.8.2103007
7. Fonseca J, Machado FVC, Santin LC, et al. Use of different reference values for handgrip strength in individuals with COPD: analysis of agreement, discriminative capacity, and main clinical implications. *J Bras Pneumol* 2022;48(5):e20210510. doi: 10.36416/1806-3756/e20210510 [published Online First: 20221114]
8. Roberts HC, Denison HJ, Martin HJ, et al. A review of the measurement of grip strength in clinical and epidemiological studies: towards a standardised approach. *Age Ageing* 2011;40(4):423–9. doi: 10.1093/ageing/afr051 [published Online First: 20110530]
9. Dag F, Guvener O, Cimen OB. Skeletal muscle architecture: is it related with aerobic capacity? *Ir J Med Sci* 2025 doi: 10.1007/s11845-025-04226-6 [published Online First: 20251220]
10. Wakayama S, Fujita Y, Fujii K, et al. Skeletal Muscle Mass and Higher-Level Functional Capacity in Female Community-Dwelling Older Adults. *Int J Environ Res Public Health* 2021;18(13) doi: 10.3390/ijerph18136692 [published Online First: 20210622]
11. Nichols S, O'Doherty AF, Taylor C, et al. Low skeletal muscle mass is associated with low aerobic capacity and increased mortality risk in patients with coronary heart disease - a CARE CR study. *Clin Physiol Funct Imaging* 2019;39(1):93–102. doi: 10.1111/cpf.12539 [published Online First: 20180830]
12. Tang D, Zhang X, Ho RW, et al. Effect of Physical Exercise on Esports Performance in First-Person Shooter Gamers: A Six-Week Randomized Controlled Trial. *Sports Med Open* 2025;11(1):162. doi: 10.1186/s40798-025-00970-2 [published Online First: 20251224]
13. Kang J, Kim S. Game over too soon: early specialization and short careers in esports. *Front Psychol* 2025;16:1585599. doi: 10.3389/fpsyg.2025.1585599 [published Online First: 20250512]
